# Brain-derived neurotrophic factor is related with adverse cardiac remodeling and high NTproBNP

**DOI:** 10.1038/s41598-019-51776-8

**Published:** 2019-10-28

**Authors:** Martin Bahls, Stephanie Könemann, Marcello R. P. Markus, Kristin Wenzel, Nele Friedrich, Matthias Nauck, Henry Völzke, Antje Steveling, Deborah Janowitz, Hans-Jörgen Grabe, Stephan B. Felix, Marcus Dörr

**Affiliations:** 1 Department of Internal Medicine B, University Medicine Greifswald, Greifswald, Germany; 2 German Centre for Cardiovascular Research (DZHK), Partner-site Greifswald, Greifswald, Germany; 3 Institute of Clinical Chemistry and Laboratory Medicine, University Medicine Greifswald, Greifswald, Germany; 4 Institute of Community Medicine, University Medicine Greifswald, Greifswald, Germany; 5 Department of Internal Medicine A, University Medicine Greifswald, Greifswald, Germany; 6 Department of Psychiatry and Psychotherapy, University Medicine Greifswald, Greifswald, Germany; 7 German Centre for neurodegenerative disease (DZNE), Partner-site Greifswald, Greifswald, Germany

**Keywords:** Biomarkers, Cardiology

## Abstract

The brain-derived neurotrophic factor (BDNF) is a neuronal growth factor essential for normal cardiac contraction and relaxation. Alterations in BDNF signaling are related to the development of cardiovascular disease. Whether BDNF is related to subclinical cardiac remodeling is unclear. We related BDNF with echocardiographic parameters and NTproBNP in a large population-based cohort (n = 2,976, median age 48 years; 45% male). Transthoracic echocardiography was performed on all subjects and BDNF was measured by ELISA. Study participants with severe kidney dysfunction, previous myocardial infarction, and LV ejection fraction <40% were excluded. Linear regression models were adjusted for age, sex, lean mass, fat mass, current smoking, systolic blood pressure and depression. Low BDNF was associated with high NTproBNP. A 10,000 pg/ml lower BDNF was related with a 2.5 g higher (95%-confidence interval [CI]: 0.2 to 4.9; p = 0.036) LV mass, 0.01 cm posterior wall thickness (0.003 to 0.022; p = 0.007) and 0.02 E/A ratio (0.003 to 0.042, p = 0.026). Here we show that low BDNF levels are related with adverse cardiac remodeling and higher levels of NTproBNP. Further research is warranted to assess if BDNF may be used to monitor neuronal-cardiac damage during CVD progression.

## Introduction

The brain-derived neurotrophic factor (BDNF) is a member of the neurotrophin family of growth factors which has been attributed to a plethora of functions including the preservation of neuronal cell viability and function as well as the prevention of neuronal degradation during stress^1^. BDNF also plays a pivotal role in non-neuronal cells. For example, BDNF is expressed in smooth muscle^2^ and endothelial cells^3^. BDNF deficiency during gestation results in murine endothelial cell apoptosis, missing intramyocardial blood vessels, microvascular leakage, thinning cardiac chambers and depressed cardiac contractility^3^. Constitutive BDNF signaling is required for physiological murine cardiac contraction and relaxation^4^. This effect of BDNF was shown to be independent of and in addition to β-adrenergic signaling where BDNF acted directly on Ca^2+^ cycling in a calmodulin-dependent protein kinase II-dependent manner.

BDNF plays a role in the progression of human cardiovascular disease. For example, this neurotrophin promotes atherogenesis and plaque instability via the activation of NAD(P)H oxidase^5^. In patients after myocardial infarction BDNF is related to inflammation and platelet activation^6^. At the same time low plasma BDNF was associated with future coronary events and mortality in 885 patients with angina pectoris^7^. In addition, BDNF levels are also reduced in heart failure patients and are inversely correlated with BNP^8^. Hence, a role of BDNF in cardiac remodeling is very likely.

Overall, previous research suggests a link between BDNF, cardiac function and cardiovascular disease. We tried to improve our understanding of this relationship by assessing the role of BDNF on left ventricular cardiac remodeling and function using data from a population based cohort from northeast Germany. We further explored the association between BDNF and the established heart failure marker N-terminal pro b-type natriuretic peptide (NTproBNP).

## Results

### Population characteristics

Median age of the study population was 48 years (range 37 to 60) and 45% were male. Median BMI was 26.7 kg/m^2^. A total of 38% were non-, 34% ex- and 28% current smokers. The prevalence of diabetes mellitus, hypertension and metabolic syndrome was 7%, 40% and 23%, respectively. The estimated glomerular filtration rate (eGFR) as an index of kidney function was within the normal range (median: 99 ml/min/1.72 mm^2^). All population characteristics and echocardiographic parameters are listed in Table 1.

**Table 1:** Population characteristics.

Parameter	Median (25^th^ and 75^th^ percentile) or %
Age (years)	48 (37, 60)
Sex (% male)	45
Systolic blood pressure (mmHg)	125 (113, 137)
BMI (kg/m2)	26.7 (23.9, 30.1)
Height (cm)	170 (163, 177)
Weight (kg)	77.8 (67.4, 88.9)
Smoking (%)	
non-smoker	38
ex-smoker	34
smoker	28
Years of schooling (%)	
<10 years	17
10 years	55
>10 years	27
Alcohol consumption (ml/day)	3.6 (0.7, 10.8)
Diabetes mellitus type 2 (%)	7
Hypertension (%)	40
Metabolic Syndrome (%)	23
eGFR (mL/min/1.73 m^2^)	99 (88, 109)
LV structural echocardiographic parameters	
LVM (g)	171 (138, 210)
LVMI (g/m^2^)	90 (77, 106)
LVD (cm)	4.9 (4.5, 5.2)
LVS (cm)	2.9 (2.6, 3.2)
PWD (cm)	0.96 (0.82, 1.07)
RWT	0.39 (0.35, 0.44)
left atrial diameter (cm)	3.8 (3.5, 4.2)
Aorta (cm)	2.7 (2.5, 3.1)
LV systolic functional echocardiographic parameters	
LVEF (%)	72 (66, 78)
Fractional Shortening (%)	41 (36, 46)
LV diastolic functional echocardiographic parameters	
MV E-wave (cm/s)	0.7 (0.6, 0.8)
MV A-wave (cm/s)	0.6 (0.5, 0.7)
E/A ratio	1.14 (0.93, 1.43)
MV duration A-wave (ms)	133 (121, 147)
MV dec. Time (ms)	179 (157, 203)
E/e′ ratio	5.8 (4.9, 7.1)
Biochemical analysis	
BDNF (pg/ml)	21,676 (17,767, 25,676)

BMI – body mass index, eGFR – estimated glomerular filtration rate, LV – left ventricle, LVM – left ventricular mass, LVMI – left ventricular mass index, LVD – left ventricular diastolic diameter, LVS – left ventricular systolic diameter, PWD – posterior wall diameter, PWT – posterior wall thickness, EF – ejection fraction, MV – mitral valve, BDNF – brain derived neurotrophic factor.

### The association BDNF and NTproBNP

**Figure 1:**
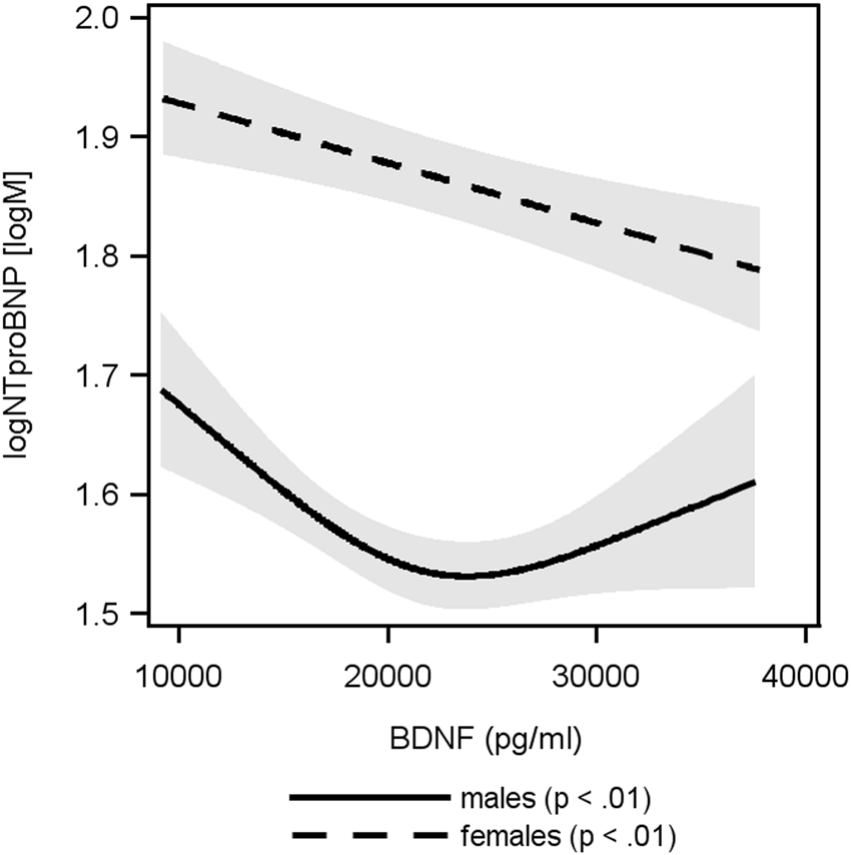
Relation between BDNF and NTproBNP. Sex specific associations between BDNF and NTproBNP. A non-linear relationship in males (p = 0.001). Women had a significantly linear inverse relationship (β coefficient −0.05 log [M] per 10,000 pg/ml higher BDNF 95%-confidence interval [CI] −0.07 to −0.02; p = 0.003). BDNF – brain derived neurotrophic factor, NTproBNP - N-terminal pro-B-type natriuretic peptide.

We found sex specific associations between BDNF and NTproBNP (Fig. 1). Specifically, we identified a non-linear relationship for males (p = 0.001) where subjects with low BDNF had the highest NTproBNP levels. With increasing BDNF NTproBNP decreased until a baseline was reached at a BDNF concentration of about 20,000 pg/ml. Study participants with a BDNF concentration between 20,000 pg/ml and 40,000 pg/ml had low levels of NTproBNP. For females, on the other hand, we identified a significant linear inverse relationship (β coefficient −0.05 log [M] per 10,000 pg/ml higher BDNF 95%-confidence interval [CI] −0.07 to −0.02; p = 0.003).

### Relation between BDNF and structural echocardiographic parameters of the left heart

**Figure 2:**
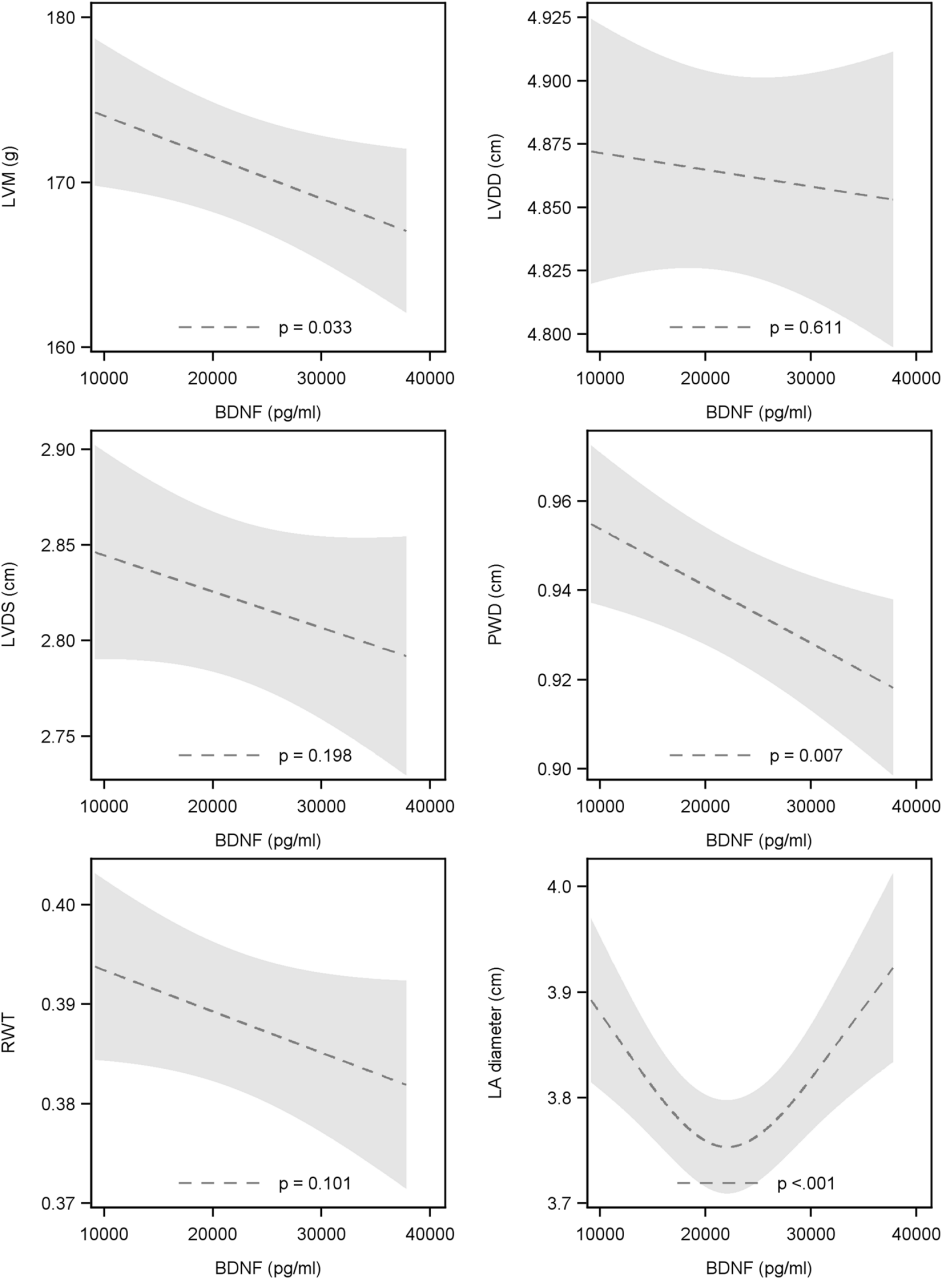
Relation between BDNF and LVM (**A**), LVDD (**B**), LVDS (**C**), PWD (**D**), RWT (**E**) as well as LA diameter (**F**). LVM – left ventricular mass, LVDD – left ventricular diastolic diameter, LVDS – left ventricular systolic diameter, PWD – posterior wall diameter, RWT – relative wall thickness, LA – left atrial diameter, BDNF – brain derived neurotrophic factor.

These results are presented in Fig. 2. BDNF was inversely related with left ventricular mass (LVM). Specifically, a 10,000 pg/ml lower BDNF was related with a 2.5 g (95%-CI: 0.2 to 4.9; p = 0.036) higher LVM and 0.01 cm (95%-CI: 0.003 to 0.022; p = 0.007) larger posterior wall thickness (PWT). Moreover, there was a non-linear relation between BDNF and left atrial diameter. Subjects with a BDNF concentration around 20,000 pg/ml had the smallest left atrial diameter. Below this concentration we observed an inverse association. Hence, low BDNF was related with a large left atrial diameter. For concentrations higher than 20,000 pg/ml a positive association was found. Thus, high levels of BDNF were also related to larger left atrial diameters. No significant relation was found for left ventricular diastolic diameter (LVDD) (−0.01 cm 95%CI: −0.02 to 0.03, p = 0.636), left ventricular systolic diameter (LVDS) (−0.02 cm 95%-CI: −0.01 to 0.05; p = 0.209) and relative wall thickness (RWT) (0.004 95%-CI: −0.001 to 0.009; p = 0.100).

### Relation between BDNF and functional echocardiographic parameters of the left ventricle

**Figure 3:**
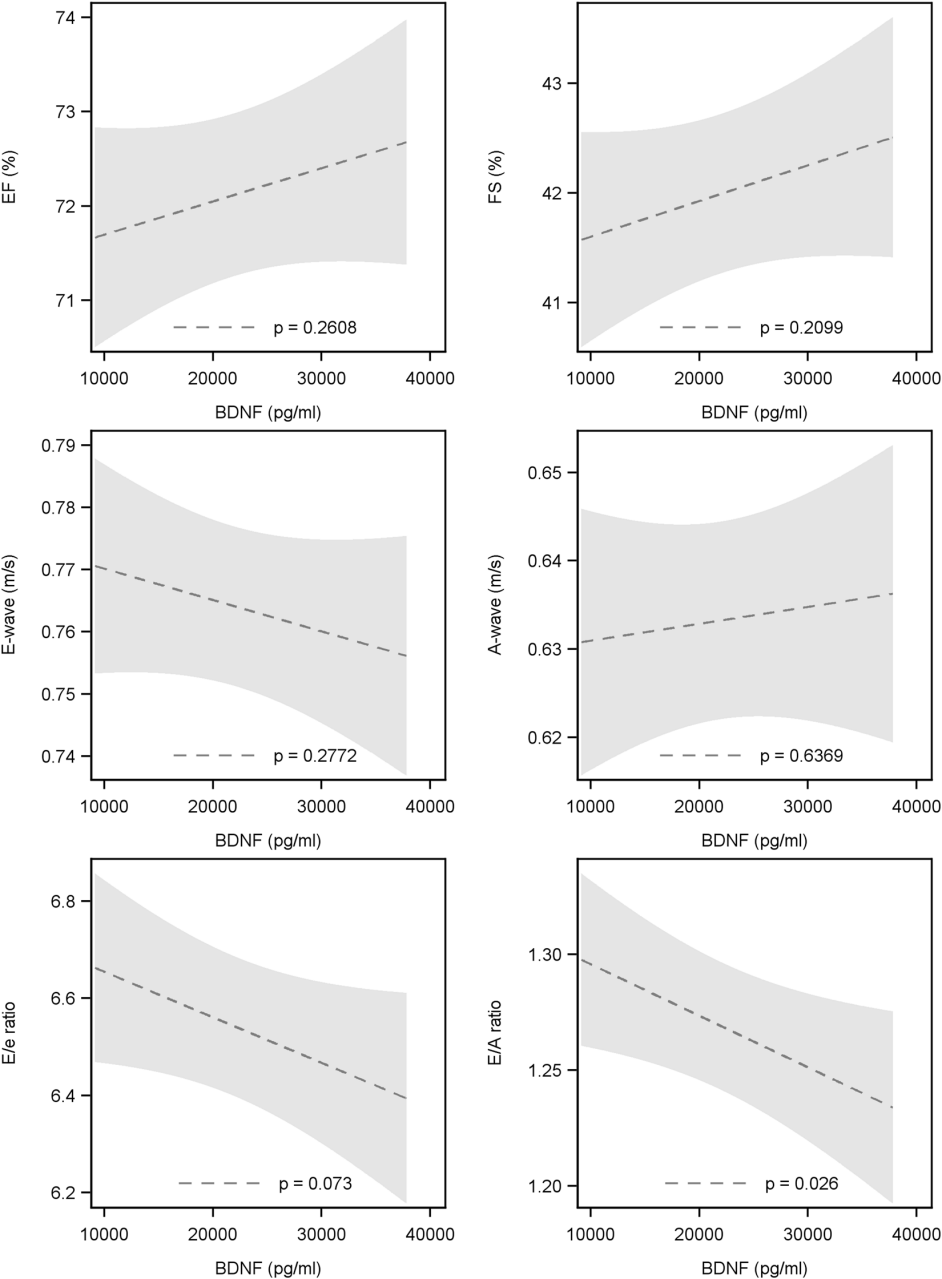
Relationship between BDNF and EF (**A**), FS (**B**), E-wave velocity (**C**), A-wave velocity (**D**), E/e ratio (**E**) as well as E/A ratio (**F**). EF – ejection fraction, FS – fractional shortening, BDNF – brain derived neurotrophic factor.

The results are presented in Fig. 3. The relation of BDNF with left ventricular ejection fraction (LVEF) and fractional shortening as parameters of systolic function showed no significant findings (LVEF: −0.35% 95-CI: −0.97 to 0.26, p = 0.261; LVFS: −0.33% 95%-CI: −0.85 to 0.19, p = 0.210). The relation of BDNF with LV diastolic function was assessed by E-wave and A-wave velocity as well as E/e′ and E/A ratio. There were no significant findings for E-wave (0.005 cm/s 95%-CI: −0.004 to 0.014, p = 0.277) and A-wave (−0.002 cm/s 95%-CI: −0.010 to 0.006, p = 0.637) velocities as well as E/e′ ratio (−0.09 95%-CI: −0.196 to 0.009 to, p = 0.073). However, low BDNF was related with a higher E/A ratio. An increase of 10,000 pg/ml in BDNF was related with 0.02 lower E/A ratio (CI: −0.042 to −0.003 to, p = 0.026).

## Discussion

Using data from a large population based study we demonstrate that low BDNF is related with higher levels of the heart failure biomarker NTproBNP and with adverse left ventricular cardiac remodeling. Recent clinical trials reported lower levels of BDNF in heart failure patients^8,9^. Hence, our results extend the current knowledge by providing evidence for a putative role of BDNF not just in heart failure but also during subclinical cardiac dysfunction and remodeling prior to manifest cardiac disease. Cardiac hypertrophy is accompanied by increased sympathetic activation leading to an upregulation of the renin-angiotensin-aldosterone system (RAAS)^10^. Brain RAAS and chronically overactive RAAS interact through positive biofeedback to synergistically maintain the diseased condition^10^. BDNF might be an internal mechanism to counteract the adverse effects of acute RAAS activation after myocardial infarction^11^. Although, in a general population rather than in a clinical setting, this study contributes to the emerging evidence featuring BDNF as a biomarker for cardio-neuronal damage during early and subclinical cardiac remodeling.

Two weeks after myocardial infarction murine BDNF levels increased in brain and plasma, but not in the heart^12^. Low BDNF in the heart as well as a knock-down of the BDNF-specific receptor, TrkB, resulted in increased fibrosis and lower ejection fraction. Moreover, intraperitoneal injection of recombinant BDNF rescued the cardiac phenotype. In patients with ischemic heart disease or acute coronary syndrome, BDNF levels are significantly lower compared to healthy controls^13,14^. In patients with unstable angina, circulating BDNF is lower when compared to stable angina and a non-coronary artery disease group^5^. These findings suggest circulating BDNF as a useful biological marker for heart disease. However, plasma BDNF, in addition, significantly positively correlates with multiple risk factors for metabolic syndrome and cardiovascular dysfunction (e.g. body mass index, fat mass, diastolic blood pressure, total cholesterol, low-density lipoprotein cholesterol, triglycerides)^15^. Thus, BDNF may lack the required specificity for a cardiac specific biomarker but might have the potential to improve our understanding of the triangular relationship between metabolic risk factors, neuronal damage and cardiac dysfunction.

An interesting and unexpected finding was the non-linear relationship between left atrial diameter and BDNF. High and low BDNF levels were related with an enlarged left atrium, while a smaller diameter was seen in subjects with BDNF levels around 20,000 pg/ml. A large left atrium is generally considered a key risk factor for atrial fibrillation (AF) and diastolic dysfunction. In mice BDNF decreases VE-cadherin cleavage to reduce atherosclerosis and promotes vascular integrity through Ets1-mediated VE-cadherin expression^16^. BDNF also prevents the TNFα induced endothelial barrier dysfunction, while TNFα can reduce BDNF expression^17^. Since the role of cadherins^18^ and TNFα^19–21^ in left atrial remodeling is well established, low levels of BDNF may increase cadherins and TNFα which in turn modulate left atrial remodeling and thereby increase AF risk. Interestingly, in the Framingham Heart Study no relation between BDNF and AF development over a 10 year time period was found^22^. The non-linear relationship between BDNF and left atrial remodeling may seem counterintuitive when compared to the other results which suggest that only low BDNF is associated with more severe adverse cardiac remodeling. However, in the Baltimore Longitudinal Study of Ageing peripheral BDNF was positively associated with several cardiovascular risk factors including body mass index and diastolic blood pressure^15^. Further, BDNF significantly increased the extent of myocardial injury in older rat hearts^23^. Based on the current literature one can only speculate whether BDNF has a protective role in response to the underlying cardiac pathophysiology or if this neurotrophin contributes to disease progression. We also previously reported a U-shaped relationship between BDNF and waist-to-hip ratio as a marker of visceral adiposity^24^. Hence, the directionality and causality between BDNF and cardiovascular disease is still undetermined and may be related to signal cross-talk. For example, BDNF and TNFα may influence each other. Future studies need to evaluate the relationship between BDNF and left atrial remodeling in specific clinical settings.

Depression and cardiovascular disorders often appear together^25^. Low BDNF levels have been reported in depressive patients^26^. Although, in our population based cohort BDNF was not associated with depression^24^. The “neurotrophic hypothesis” for depression even postulates that a stress-induced decrease in BDNF initiates depression^27,28^. As mentioned above, BDNF is also related to cardiovascular disease. In a large population-based cohort low BDNF was associated with higher mortality and incidences of cardiovascular events^29^. Since BDNF is related to depression and cardiovascular disease, one may speculate that this neurotrophin influences both conditions^30^, potentially through inflammation^31^ since patients with depression^32^ and acute coronary syndrome^33^ are characterized by increased inflammation. This hypothesis is additionally supported by the fact that inflammation can decrease BDNF^34^. Nonetheless, we used major depressive symptoms as a confounder in our analysis and BDNF was still significantly associated with adverse cardiac remodeling and NTproBNP. Hence, low BDNF may be associated with higher cardiac risk independent of depression.

The findings of our analysis need to be interpreted in the context of several limitations. First, we only included Europeans living in rural areas in our sample. Hence, we do not know if our findings are true for other ethnicities, children or elderly age-groups. Second, we only had access to cross-sectional data from one time point. Thus, we do not know whether a decrease in circulating BDNF over time is associated with adverse left ventricular structure and function. Third, although we used a directed acyclic graph to identify metabolic, cardiovascular and psychiatric confounders for our multivariable models, we cannot exclude the possibility of further residual confounding. Fourth, we only used two questions to determine whether subjects ever experienced a major depressive disorder. This may be less sufficient to account for the complexity of depressive disorders. Nonetheless, strengths of our study are the population-based setting, the large number of study participants, the use of standardized data collection methods, the capacity to perform adjustment for a variety of clinical, behavioral and psychiatric risk factors and the availability of a standardized echocardiographic examination from a large number of participants.

In summary, this study revealed that subjects with low BDNF have higher NTproBNP levels and adverse early and subclinical left ventricular remodeling. Further, we provided evidence of a non-linear relationship between BDNF and left atrial alterations. We showed that these findings are independent of depressive symptoms. Taken together our results might support the notion that BDNF could be a marker for neuronal-cardiac damage during the early phases of cardiac remodeling.

## Materials and Methods

### Study population

The Study of Health in Pomerania (SHIP) is a prospective population-based cohort of adults from West Pomerania, a north-eastern region in Germany of approximately 220,000 inhabitants. The first sample (SHIP-0) was surveyed between 1997 and 2001 using a stratified cluster-random sample of 7,008 individuals. The net sample (without migrated or deceased persons) included 6,265 eligible individuals. A total of 4,308 (2,192 women) subjects participated (response: 68.8%) in SHIP-0^35^. The data used in the presented analysis were derived from SHIP-Trend, a cohort initiated ten years after SHIP-0 in the same region^36^. In brief, from the total population of West Pomerania, a rural area in the northeastern part of Germany, a two-stage stratified cluster sample of 8,016 adults between the ages of 20–79 years was drawn. In total, 4,420 individuals participated in the study (response of 50.1%). Data used in this analysis is based on data collected during the baseline examination of SHIP-TREND which took place between 2008 and 2011. We have previously used SHIP-TREND to assess the relation between BDNF and BMI^24^. The study was approved by the ethics committee of the University of Greifswald, complies with the Declaration of Helsinki and all study participants gave written informed consent. SHIP data are publically available for scientific and quality control purposes. Data usage can be applied for via www.community-medicine.de.

For the present analysis individuals with severely impaired renal function (estimated glomerular filtration rate [eGFR] <30 mL/min/1.73 m^2^), previous myocardial infarction, left ventricular ejection fraction <40%, atrial fibrillation, extreme values for BDNF and NTproBNP (below 1^st^ percentile, higher 99^th^ percentile) and missing data were excluded. The total sample size was 2,976 subjects.

### Interview, medical and laboratory examination

Trained and certified staff used standardized computer-assisted interviews to ask the patients about their age, sex, years of schooling and smoking habits. Smoking was classified as current smoker, nonsmoker or former smoker. All participants underwent an extensive standardized medical examination. Anthropometric measurements included height and weight based on recommendations of the World Health Organization (WHO)^37^. Body mass index (BMI) was calculated by dividing weight (kg) by height (cm) to the square. Diabetic patients were identified based on the self-reported use of antidiabetic medication (anatomic, therapeutic, and chemical (ATC) code: A10) in the last 7 days or a glycosylated hemoglobin level >6.5%. Blood pressure (BP) was assessed after a 5 min resting period in sitting position. Systolic and diastolic BP were measured three times, with three minutes rest in between, on the right arm using a digital blood pressure monitor (HEM-705CP, Omron Corporation, Tokyo, Japan). The average of the second and third reading was used. Hypertensive patients were identified by either self-reported antihypertensive medication (ATC: C02, C03, C07, C08 and C09) or a systolic BP above 140 mmHg and/or a diastolic value of more than 90 mmHg. Lean mass and fat mass were measured by bioelectrical impedance analysis (BIA) using a multifrequency Nutriguard-M device (Data Input, Pöcking, Germany) and the NUTRI4 software (Data Input, Pöcking, Germany) in participants without pacemakers. The electrodes were placed on hand and wrist as well as ankle and foot. The test frequency was measured at 5, 50 and 100 kHz following the manufacturer’s instructions^38^. Body mass index (BMI) was calculated by dividing weight (kg) by height (cm) to the square. Diabetic patients were identified based on the self-reported use of antidiabetic medication (anatomic, therapeutic, and chemical (ATC) code: A10) in the last 7 days or a glycosylated hemoglobin level >6.5%. Blood pressure (BP) was assessed after a 5 min resting period in sitting position. Systolic and diastolic BP were measured three times, with three minutes rest in between, on the right arm using a digital blood pressure monitor (HEM-705CP, Omron Corporation, Tokyo, Japan). The average of the second and third reading was used. Hypertensive patients were identified by either self-reported antihypertensive medication (ATC: C02, C03, C07, C08 and C09) or a systolic BP above 140 mmHg and/or a diastolic value of more than 90 mmHg. Metabolic syndrome was defined by any three or more of the five components proposed by ATP III^39^ and updated with minor modifications by the American Heart Association and the National Heart, Lung, and Blood Institute^40^ and were modified for the use of nonfasting blood samples^41^: (1) abdominal obesity, waist circumference ≥104 cm in men and ≥88 cm in women; (2) elevated triglycerides, >2.0 mmol/l or lipid medication (ATC code C10ab); (3) low HDL cholesterol, <1.03 mmol/l in men; (4) high blood pressure, >130/85 mmHg or antihypertensive medication (ATC code C02); (5) high blood glucose, >8.0 mmol/or diabetes medication (ATC code A10). The electrodes were placed on hand and wrist as well as ankle and foot. The test frequency was measured at 5, 50 and 100 kHz following the manufacturer’s instructions^38^. Alcohol consumption (in grams per day) was derived from a beverage-specific quantity-frequency index.

Major depressive disorder (MDD) and recurrent MDD were diagnosed according to DSM-IV using the Munich-Composite International Diagnostic Interview (M-CIDI)^42–44^. The screening questions for depressive disorders comprised the following two items: “Feelings of sadness or depressed mood for a period of at least 2 weeks” and “Lack of interest, tiredness, or loss of energy for a period of at least 2 weeks”.

A non-fasting venous blood sample was drawn from all subjects in supine position (between 7 am and 4 pm). The eGFR was calculated according to Levey *et al*. (eGFR = 186 × (plasma creatinine concentration × 0.0113118)^−1.154^ × age^−0.203^) multiplied by 0.742 for female subjects and expressed as mL/min/1.73 m^2,45^. BDNF and NTproBNP were measured according to the manufacturer’s recommendations using an ELISA (R&D Systems Europe, UK) and Dimension Vista (Siemens, Germany), respectively.

### Ultrasound measurements

Two-dimensional, M-Mode and Doppler echocardiography were performed using the Vivid-I system (GE Medical Systems, Waukesha, USA) as described in detail elsewhere^46^. Measurements of LV end-diastolic and end-systolic diameter (LVDD, LVDS) and septal as well as posterior wall thickness (SWT, PWT) were performed according to the guidelines of the American Society of Echocardiography^47^. LV mass (LVM) was calculated according to the formula: LVM (g) = 0.8 × (1.04 × ((LVDD + SWT + PWT)^3^ - LVDD^3^)) + 0.6 g as described by Devereux and Reichek^48,49^. LVM was indexed (LVMI) for body surface area (BSA) according to Duboi (BSA = 0.20247 × height (m)^0.725^ × weight (kg)^0.425^)^50^, which linearizes the relations between LVM and height and identifies the impact of obesity. LV wall thickness (WT), relative wall thickness (RWT), LV ejection fraction (EF) and fractional shortening (FS) were calculated following the formulas below according to the guidelines of the American Society of Echocardiography^51^. Transmitral pulsed-wave Doppler was used to record early (E) and late (A) wave ventricular filling velocities. Certification examinations for inter-observer variations revealed an agreement of >90%^46^.

### Statistics

Continuous data are expressed as median and 25^th^/75^th^ percentile. Nominal data are expressed as percentages. Differences between groups were calculated using Kruskal-Wallis (continuous variables) and χ^2^ test (nominal variables), respectively. A linear regression adjusted for sex, age, fat mass, lean mass, systolic blood pressure, current smoking and depression was fitted to relate BDNF the echocardiographic parameters of interest. Furthermore, restricted cubic splines^52^ were used to detect possible nonlinear dependencies of BDNF on the investigated echocardiographic parameters. Three knots were pre-specified, located at the 5^th^, 50^th^, and 95^th^ percentiles^52^, resulting in one component of the spline function. Potential sex specific associations between BDNF and echocardiographic parameters were assessed by adding the appropriate interaction term into the model. If this term was significant the analysis was stratified by sex. A p < 0.05 was considered statistically significant. All statistical analyses were performed in SAS 9.4 (SAS Institute Inc., Cary, NC, USA). All parameters are reported as median and 25^th^ as well as 75^th^ percentile unless indicated otherwise. All results of the linear regression analysis are given as a 10,000 ng/ml decrease in BDNF.

### Ethics approval and consent to participate

All participants provided written informed consent and SHIP was approved by the Ethics Committee of the University Medicine Greifswald.

## Data Availability

SHIP data are publically available and can be applied for at www.community-medicine.de.
